# Clinical Effectiveness of Arthroscopy-Assisted Fixation in the Treatment of Avulsed Posterior Cruciate Ligament Injuries

**DOI:** 10.7759/cureus.50152

**Published:** 2023-12-08

**Authors:** Vasant Gawande, Ankit Badge

**Affiliations:** 1 Orthopedics, Datta Meghe Medical College, Datta Meghe Institute of Higher Education and Research, Nagpur, IND; 2 Medicine, Datta Meghe Medical College, Datta Meghe Institute of Higher Education and Research, Nagpur, IND

**Keywords:** advancements, concomitant injuries, rehabilitation, clinical effectiveness, arthroscopy-assisted fixation, avulsed posterior cruciate ligament injuries

## Abstract

Avulsed posterior cruciate ligament (PCL) injuries are complex orthopedic challenges that require careful consideration and optimal management. Arthroscopy offers advantages, including smaller incisions, reduced soft tissue disruption, reduced postoperative pain, and improved visualization of intraarticular anatomy. Arthroscopy-assisted fixation results in superior clinical outcomes. Patient-specific factors, graft choice, and timing of surgery significantly impact outcomes. Rehabilitation is vital and requires a tailored approach to restore knee function. Biomechanically, arthroscopy-assisted fixation enhances joint stability and range of motion, reducing the risk of secondary injuries. Advancements in technology and surgical techniques further improve outcomes. Concomitant injuries and incorporation are essential considerations. Arthroscopy-assisted fixation is a recommended approach, but personalized care is crucial for successful recovery. Its precision in reattaching the PCL enhances joint stability and clinical results, aligning with outcomes seen in conventional procedures. Using biocompatible materials in fixation devices has significantly reduced the risk of allergic reactions or complications. This has allowed a faster and smoother recovery process for patients undergoing arthroscopy-assisted fixation. The incorporation of physical therapy and rehabilitation programs after surgery plays a vital role in restoring joint function and preventing muscle atrophy. The combination of advanced technology, surgical techniques, and personalized care has greatly improved the success rate of arthroscopy-assisted fixation procedures. Advancements in technology further improve patient outcomes, but each case should be individually assessed to determine the most appropriate treatment approach.

## Introduction and background

An injury to the posterior cruciate ligament (PCL) avulsion is uncommon, and reconstruction is challenging because of the complex structures and complicated reconstruction techniques. Treatment of these injuries has been significantly transformed, with arthroscopy-assisted fixation becoming a popular and promising technique [1]. Arthroscopy-assisted fixation has significant benefits compared to conventional open surgical procedures. It reduces soft tissue disturbance, resulting in smaller incisions, reduced postoperative pain, and faster recovery durations [2]. Improved intraarticular anatomical visualization makes it possible to precisely restore PCL, improving joint stability, and possibly improving therapeutic results. The capacity to return to activities and patient satisfaction are frequently improved [3]. Long-term problems include graft failure, joint stiffness, and the possibility of osteoarthritis. Short-term consequences include postoperative swelling, discomfort, and restricted range of motion. Age, sex, and severity of injury are patient-specific characteristics that significantly affect surgical results, necessitating individualized treatment strategies [4]. Arthroscopy-assisted fixation and rehabilitation are essential for recovery. A planned program adapted to the requirements of each patient involves sport-specific proprioception, muscle strengthening, wound repair, and pain reduction exercises. Successful rehabilitation is largely dependent on patient cooperation, the complexity of the injury, and professional guidance [5]. Arthroscopy-assisted fixation improves the range of motion, reduces abnormal joint movements, and restores knee stability from a biomechanical perspective. Restorative maintenance of knee function is necessary to return to normal activities and reduce the risk of subsequent injuries [6]. Improvements in surgical techniques, graft materials, and arthroscopy technology have contributed to the effectiveness of this approach. Advanced imaging methods that help preoperative planning and intraoperative efficacy include fluoroscopy and magnetic resonance imaging (MRI) [7]. Treatment plans and results are significantly influenced by simultaneous injuries and the timing of surgery [8]. The normal graft is incorporated, an important aspect of therapy success, and is affected by joint stability, range of motion, and joint strength. Patient experiences and satisfaction levels differ depending on the type of graft used, highlighting the need for a customized approach [9]. This review investigates the clinical effectiveness of arthroscopy-assisted fixation in the treatment of avulsed PCL injuries.

## Review

Methodology

To conduct a comprehensive literature search, we use the PubMed and Google Scholar advanced search strategy to obtain articles from PubMed and Google Scholar using the following terms: ((((((((((((((((((((Posterior Cruciate Ligament Injuries) OR (posterior cruciate ligament Injury)) OR (PCL injury)) OR (PCL injuries)) AND (Arthroscopy)) OR (Fixation Techniques)) OR (Arthroscopy-Assisted Fixation)) AND (Treatment Outcomes))) OR (Effectiveness)) OR (Rehabilitation)) AND (Advancements).

Articles Screened

**Figure 1:**
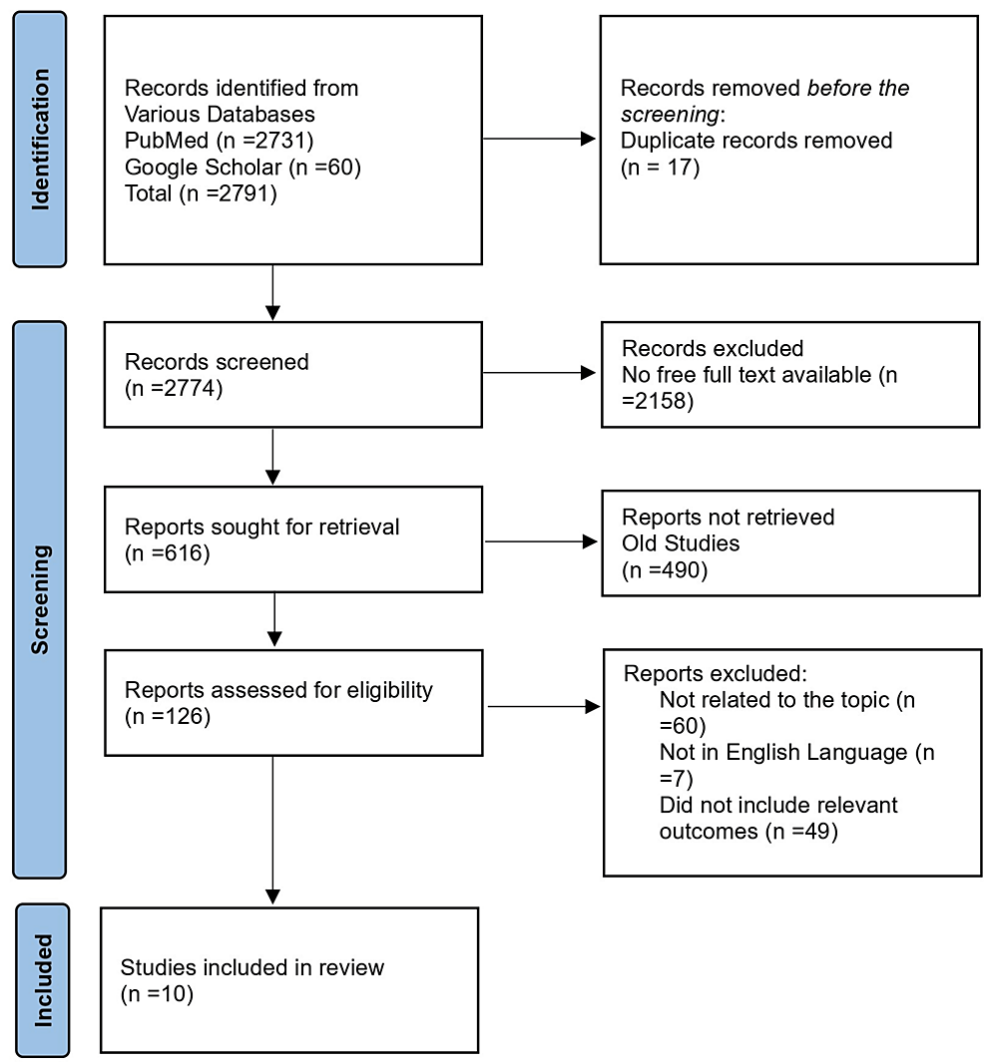
PRISMA flowchart. n, number of studies; PRISMA, Preferred Reporting Items for Systematic Reviews and Meta-analyses

Based on relevancy, research design, and publication date, filter publications were selected. We excluded studies unrelated to the treatment of avulsed PCL injuries. After an initial screening of 2,791, we eliminated 17 duplicates. After the final screening of 2,774 articles, we excluded 2,648 studies that were not available in free full text and those that were considered outdated. After excluding 116 articles that were unrelated to the topic, were unavailable in the English language, and did not include relevant outcomes, we included 10 studies for the final review from 2020 to 2023 (Figure 1).

The articles included in the review describe arthroscopy-assisted fixation techniques for a knee injury (Table 1).

**Table 1:** Literature review. PCL, posterior cruciate ligament; ACL, anterior cruciate ligament; BMI, body mass index

Serial no.	Author	Year	Insights	Conclusion
1	Kew et al. [10]	2022	Treatment outcomes and advancements in rehabilitation for PCL injuries	Conservative treatment of isolated PCL injuries has excellent outcomes and return to play. Rehabilitation plays a critical role in the outcome after PCL injury and the ability to return to athletics.
2	Oganesyan et al. [11]	2022	Complete PCL injuries were found to occur at a higher age in females, with an age-dependent distribution of the location of the PCL injury and a number of accompanying injuries, as well as significant negative correlations between age and associated ACL injuries.	Sex differences in the accompanying injuries with PCL tears. Complete PCL injuries occur at a higher age in females.
3	Kaarre et al. [12]	2022	Explore patients' characteristics and injury profiles for ACL and PCL injuries, providing valuable information for diagnosis and treatment planning.	Females, older patients, and patients with a higher BMI were more susceptible to complex injuries such as combined PCL injuries. ACL injuries were more common in men and were sustained during pivot sports or alpine skiing.
4	Piedade et al. [13]	2023	The PCL tibial inlay reconstruction technique was presented using a set of instruments that involved three tools (a slot cut, a bone plug positioner, and an impacter).	The PCL tibial inlay reconstruction technique using a set of instruments allows for a more reproducible posteromedial approach. The technique involves three tools (a slot cut, a bone plug positioner, and an impacter) for producing a PCL tibial slot in a PCL inlay reconstruction with the patient supine in the reconstruction of bicruciate ligament injury.
5	Shubert et al. [14]	2022	The use of needle-arthroscopy-assisted arthroscopic PCL reconstruction for optimal visualization of the PCL tibial footprint eliminates the need for multiple arthroscopes	Needle arthroscopy-assisted arthroscopic PCL reconstruction improves visualization. Needle arthroscopy eliminates the need for multiple arthroscopes.
6	Ng et al. [15]	2021	Isolated PCL injuries can often be treated nonoperatively with rehabilitation, but there is controversy over the best reconstructive method for chronic injuries.	Most isolated PCL injuries can be treated non-operatively. PCL reconstructions are performed in chronic injury.
7	Lamb et al. [16]	2021	PCL injuries are rare in pediatric populations and there are relatively little data on the epidemiology, natural history, and optimal management of these injuries, as mentioned in this paper.	PCL injuries are rare in pediatric populations. Optimal management is crucial to preserve knee function.
8	Gupta et al. [17]	2021	Arthroscopic reconstruction of isolated PCL and combined ACL and PCL tears showed similar functional results.	There are no differences in functional outcomes between patients with isolated PCL and patients with combined ACL and PCL reconstruction.
9	Vermeijden et al. [18]	2020	Primary repair of proximal and distal PCL tears may be an alternative treatment to reconstruction, preserving native tissue and maintaining proprioception.	Primary repair may be an alternative to PCL reconstruction. Appropriate patient selection is crucial to success.
10	Razi et al. [19]	2020	Advancements in arthroscopy-assisted fixation techniques have improved treatment outcomes and rehabilitation for PCL injuries.	Advances in surgical techniques have made PCL reconstruction promising. Surgical treatment is recommended for isolated PCL tears.

Two studies [10,11] found the success of conservative treatment for isolated PCL injuries, with rehabilitation for long-term results, and explored relationships between age and anterior cruciate ligament (ACL) injuries, highlighting age-dependent distribution and accompanying injuries in women with PCL tears. One study [12] explores patient characteristics and injury profiles, identifying susceptibility factors for complex injuries. Two studies [13,14] present surgical techniques for tibial inlay reconstruction and needle-arthroscopy-assisted arthroscopic PCL reconstruction to improve reproducibility and visualization. Two studies [15,16] discuss the controversy over reconstructive methods for chronic PCL injuries, advocating for nonoperative treatment in most cases, highlighting the rarity of PCL injuries in pediatric populations, and stressing the importance of optimal treatment to preserve knee function. One study [17] found similar functional results in the arthroscopic reconstruction of isolated PCL and combined ACL and PCL tears. One study [18] proposes primary repair as an alternative to reconstruction, preserving native tissue and proprioception with appropriate patient selection. Advancements in arthroscopy-assisted fixation techniques have improved results, and a study recommends surgical treatment for isolated PCL tears [19].

The clinical efficacy of arthroscopy-assisted fixation over traditional open surgeries in the treatment of avulsed PCL injuries has become a major interest in orthopedic surgery [20]. Arthroscopy-assisted fixation has several possible benefits compared to conventional surgical techniques [21]. The benefits of the minimally invasive approach include smaller incisions, less disturbance of soft tissue, and less pain after surgery, which can result in faster recovery [22]. Furthermore, arthroscopic procedures improve intraarticular anatomical visualization, helping to achieve more accurate PCL reattachment and identification [14]. Arthroscopy-assisted graft placement and fixation can result in greater restoration of PCL function and joint stability, reducing the risk of postoperative complications and long-term joint deterioration [23]. Patient satisfaction and earlier return to activities have also been observed in patients who completed arthroscopy-aided surgeries [24]. Based on the severity of the injury, surgeon experience, and patient-specific conditions, the results of arthroscopy-assisted fixation in avulsed PCL injuries can vary [25]. Traditional open surgeries have a longer history of favorable developments and may be preferred in rare cases, despite being associated with larger incisions and potential soft tissue trauma [26].

Advantages of arthroscopy-assisted PCL fixation

Compared to alternative surgical procedures, the arthroscopy-assisted fixation approach for avulsed PCL injuries significantly improves postoperative knee stability and functional outcomes [27]. For avulsed PCL injuries, arthroscopy-assisted fixation offers several important advantages. It enables improved reattachment and restoration of anatomical alignment by precisely visualizing the PCL and its attachment site. This stimulates improved joint stability and prevents the tibia from translating posteriorly when performing different activities [28]. Compared to open surgery, this minimally invasive technique results in smaller incisions and less soft tissue stress, reducing postoperative pain and swelling [29]. Arthroscopy-assisted fixation also tends to allow faster initiation of postoperative rehabilitation, promoting improved joint mobility, muscle strength, and proprioception. This contributes to better long-term functional outcomes [30]. Additionally, the reduced risk of complications, such as infection and wound healing issues, improves overall patient satisfaction and functional recovery. This technique may offer the advantage of a faster return to sports and high-demand activities for athletes and active individuals due to improved stability and rapid rehabilitation [31].

Complications in arthroscopy-assisted PCL fixation

Arthroscopy-assisted fixation for avulsed PCL injuries offers several advantages but is not without potential complications, varying in severity and duration [32]. Short-term complications, such as infections (rare due to the minimally invasive nature of arthroscopy), postoperative swelling, pain, and temporary limitations in range of motion, are relatively common but generally milder than traditional open techniques [27,33]. Long-term complications can include graft failure or loosening, which can affect both approaches and joint stiffness, with arthroscopy potentially offering some mitigation due to its minimally invasive nature [34]. The risk of osteoarthritis development is a concern for both methods and depends on factors such as surgical precision and postoperative care. Although rare, nerve or vascular injuries can occur in open surgical techniques, with arthroscopy-assisted surgical techniques generally having low associated risk [4].

Influential patient factors in PCL fixation outcomes

Patient-specific factors are crucial in shaping the outcomes of arthroscopy-assisted fixation for avulsed PCL injuries [35]. Elements such as age, sex, and injury severity significantly influence the success of surgery and the recovery process. Age plays an important role, with younger patients benefiting from improved healing potential, but potentially exposing the repaired ligament to greater stress due to more demanding activities [36]. On the contrary, older patients may face challenges in tissue healing and overall joint function. Gender differences complicate ligament laxity and recovery rates [37]. Injury severity, a crucial determinant, can complicate surgery and postoperative stability, especially in multiple ligament injuries [38]. The timing of surgery also matters, and early intervention often offers advantages. Recognizing this multifaceted patient-specific factor is important to tailor treatment strategies and manage expectations to optimize surgical outcomes and patient recovery [39].

Rehabilitation

Rehabilitation plays a critical role in the recovery of patients with avulsed PCL injuries following arthroscopy-assisted fixation. The rehabilitation program is carefully phased to optimize joint stability and functional restoration [10]. In the early postoperative phase of PCL injury, immediate care focuses on wound healing, pain management, and swelling control while gently introducing a range of motion exercises to prevent joint stiffness [40]. Moving into the mid-term phase, attention turns to muscle strengthening, particularly the quadriceps and hamstrings, to support joint stability. Proprioception and balance training enhance joint awareness and control [41]. The late-term phase introduces sport-specific activities, plyometrics, and agility training to replicate sports demands and improve performance. The return to sports is gradual and depends on individual progress. Long-term maintenance involves ongoing strength and conditioning exercises to maintain joint stability. Please keep regular monitoring and follow-up appointments to ensure that they continue and address any concerns. Patient compliance, the severity of the injury, surgical quality, and professional direction are important components of successful rehabilitation [10,42].

Biomechanical and kinematics impact

The biomechanics and kinematics of the knee joint in patients with avulsed PCL injuries have been significantly modified by arthroscopy-assisted fixation, which is essential to reestablish full knee function [43]. By reattaching the avulsed ligament, biomechanics and kinematics are restored, ensuring joint stability, which is a crucial function of the PCL. This improvement in strength enhances confidence during weight-bearing exercises and reduces the possibility of injuries caused by instability [44]. Excessive posterior tibial translation during knee flexion, which occurs in PCL injuries, is limited by arthroscopy-assisted fixation and promotes natural knee kinematics by restoring proper PCL tension and function [45]. It also supports optimal rotational stability, enabling simpler and more comfortable joint motions. In addition to biomechanical advantages, arthroscopy-assisted fixation improves knee function [46]. Biomechanics and kinematics enhance flexibility and ease of movement throughout daily and athletic activities and range of motion. Functional exercises such as running, walking, jumping, and moving require proper knee biomechanics and kinematics, and arthroscopy-assisted fixation provides smoother, more controlled joint movement during these activities [47].

Restoring PCL function also helps prevent secondary injuries due to altered joint mechanics, such as meniscal tears, cartilage damage, and early-onset osteoarthritis [36]. Restoration of knee biomechanics and kinematics is important to facilitate a successful return to sports and high-demand activities, reducing the risk of re-injury and allowing athletes to perform at their pre-injury level [48]. Patient-related factors in arthroscopy-assisted PCL fixation are described in Table 2.

**Table 2:** Patient-related factors in arthroscopy-assisted PCL fixation. Sources: [43-47]. PCL, posterior cruciate ligament

Aspects	Description
Restoration of joint stability	Reattaches the avulsed ligament to restore joint stability, a crucial function of the PCL
Reduction of posterior tibial translation	Limits excessive posterior tibial translation during knee flexion, reducing instability
Restoration of proper tension and function	Restores proper PCL tension and function, promoting natural knee kinematics
Improved rotational stability	Supports optimum rotational stability, enabling smoother and more comfortable joint movements
Enhanced flexibility and range of motion	Improves flexibility and ease of movement in daily and athletic activities, improving range of motion
Prevention of secondary injuries	Prevents secondary injuries due to altered joint mechanics, reducing the risk of problems such as meniscal tears
Facilitating a successful return to sports	Enables a successful return to sports and high-demand activities, reducing the risk of re-injury

Cost-effectiveness of PCL injury fixation

The cost-effectiveness of arthroscopy-assisted fixation compared to other surgical methods for avulsed PCL injuries depends on hospitalization time, surgical procedure costs, rehabilitation, long-term follow-up, return to work and activities, and patient satisfaction [49]. Arthroscopy-assisted fixation tends to involve shorter hospital stays due to its minimally invasive nature, potentially reducing hospital expenses. However, there may be higher surgical costs due to specialized equipment. Rehabilitation costs can vary according to the intensity and duration of sessions [22]. Long-term follow-up can benefit from improved biomechanics and kinematics, potentially reducing complications. Furthermore, improved quality of life and patient satisfaction can indirectly impact cost-effectiveness by reducing downstream healthcare costs [50].

Advancements in arthroscopy for PCL injuries

Advancements in arthroscopic technology and surgical techniques have significantly improved the effectiveness of arthroscopy-assisted fixation for avulsed PCL injuries [51] (Table 3).

**Table 3:** Advancements in arthroscopic technology and surgical techniques. 3D, three-dimensional; PCL, posterior cruciate ligament; MRI, magnetic resonance imaging

Advancements	Description
Improved visualization	The modern arthroscopic technique includes high-definition 3D imaging, which is beneficial for better visualizing the avulsed PCL fragment, its orientation, and the surrounding structures. Enhanced visualization helps to accurately plan and execute the repair [52].
Minimally invasive approaches	Arthroscopy is a minimally invasive procedure, and progress has made it even less invasive. Smaller incisions and upgraded instruments reduce tissue damage, leading to faster recovery and fewer postoperative complications [22].
Advanced suturing techniques	Advanced arthroscopic suturing techniques and devices enable surgeons to precisely reattach the avulsed PCL to its anatomical attachment point. These techniques are less traumatic to ligament tissue and promote faster recovery [53].
Biocompatible fixation materials	The use of biocompatible materials for fixation has become more common, reducing the risk of complications and the need for additional surgeries to remove hardware [54].
Better rehabilitation protocols	Advancements in surgical techniques have led to more effective postoperative rehabilitation protocols. This protocol ensures that patients regain strength and range of motion in a controlled manner, promoting better long-term outcomes [55].
Patient-specific approaches	Arthroscopic procedures now incorporate patient-specific planning and 3D printing technology, allowing a tailored approach to each patient and improving repair precision [56].
Enhanced knowledge and training	As arthroscopic technology has evolved, so has the training of orthopedic surgeons. Surgeons have better educational resources and simulation tools, leading to improved surgical skills and expertise [57].
Outcome assessment	Advanced imaging techniques, including MRI and ultrasound, allow surgeons to assess the success of the repair with greater accuracy. This ensures that issues can be detected and addressed, leading to better long-term results [58].

Factors affecting patient-reported outcomes

Patient-reported outcomes in arthroscopy-assisted fixation for avulsed PCL injuries reveal notable benefits. Patients frequently report a substantial reduction in post-operative pain thanks to the minimally invasive nature of this surgical approach, which involves smaller incisions [59]. Improved joint stability is a commonly cited advantage, enhancing patient confidence in everyday activities and sports participation. Enhanced knee functionality and expanded range of motion are often reported, positively influencing patients' abilities to engage in routine activities, including walking, running, and sports. Improvement in physical capabilities contributes to an overall increase in their quality of life, allowing patients to participate in activities to avoid before surgery [48]. The patient-reported outcome collectively reflects the positive impact of arthroscopy-assisted PCL fixation on individuals' lives [23].

Arthroscopy, a minimally invasive surgical technique, is a differentiating characteristic from traditional open surgery. Arthroscopy has many benefits, including reduced postoperative pain, fast recovery times, and smaller and less noticeable scars. These factors often result in higher patient satisfaction levels with the arthroscopic approach [22]. On the contrary, conservative management, which involves nonsurgical methods such as physical therapy, can be effective for certain PCL injuries. However, it may sometimes provide a different level of stability and functional restoration than arthroscopic fixation [42]. Another crucial aspect to consider in PCL reconstruction is the choice of graft material. Surgeons use graft options, such as autografts (tissue harvested from the patient's body) or allografts (donor tissue). The selection of graft type can significantly influence treatment success [60]. Patient experiences and satisfaction levels can vary depending on the type of graft used, highlighting the importance of a personalized approach to the treatment of PCL injury [45].

Critical impact of timely arthroscopic fixation in PCL injuries

The timing of arthroscopy-assisted fixation relative to the initial injury is critical in determining the overall healing process, functional recovery, and long-term outcomes of avulsed PCL damages [20]. Swift intervention offers several advantages, typically within the acute phase after injury. Early surgery enables a more precise and direct repair of the avulsed ligament, promoting better anatomical alignment and healing. This results in improved joint stability, reduced risk of secondary damage, and a more favorable environment for tissue recovery [61]. Timely treatment also positively influences functional recovery by preventing joint stiffness, muscle weakness, and compensatory movements that can impede rehabilitation. Patients who undergo early intervention often enjoy faster and more complete restoration of joint function, range of motion, and strength. Long-term and timely resolution of avulsed PCL injuries promptly contributes to sustained joint health and stability, minimizing the risk of degenerative changes. By reducing the duration of functional compromise, patients are more likely to regain their levels of activity levels, improving their overall quality of life [62]. However, the decision for arthroscopy-assisted fixation should be made in consultation with a thorough clinical evaluation, considering the severity of the injury. In some cases, delaying surgery to allow for reduced swelling and improved soft tissue condition may be advisable [63].

Impact of concomitant injuries

Concomitant injuries, such as meniscal tears or collateral ligament damage, significantly influence the treatment strategy and outcomes of arthroscopy-assisted fixation for avulsed PCL injuries [64]. The treatment strategy becomes more complex when avulsed PCL injuries coincide with other knee injuries. Surgeons must meticulously assess the extent and severity of all injuries to determine priorities. This may involve simultaneous repair of multiple injuries or a staged approach, with severe meniscal tears or collateral ligament injuries often taking precedence [27]. Concomitant injuries also impact surgical planning, affecting the type and timing of surgery and the choice of graft material for PCL repair. Rehabilitation following arthroscopy-assisted fixation can be more complex when multiple injuries are present, requiring customized protocols to address specific needs. Concomitant injuries can influence outcomes, potentially improving joint stability, function, and pain relief, prolonging recovery times, and affecting the ability to return to high-impact activities. Coordination between orthopedic surgeons, physical therapists, and specialists is crucial to a complete and successful recovery [65].

Significance of graft incorporation

Incorporation of the graft and healing rate at the fixation site following arthroscopy-assisted treatment of avulsed PCL injuries is a critical determinant of treatment success. This process involves integrating the graft material into the native PCL insertion point and typically occurs several months to a year after surgery [60]. Successful graft incorporation is strongly correlated with improved functional outcomes, including improved stability of the knee joint, increased range of motion, and greater strength in the affected leg. These improvements are vital for resuming daily activities and sports participation [66]. Furthermore, the incorporation of the graft contributes to the long-term stability of the knee joint, reducing the risk of recurrent instability and potential future knee problems. Patient adherence to rehabilitation plans and choice of graft material and surgical technique influence the incorporation of the graft [67].

Challenges and considerations

Implementing arthroscopy-assisted fixation for avulsed PCL injuries presents distinct challenges in rehabilitation. Specific considerations are crucial to optimizing patient recovery. The primary challenge lies in allowing ample time for the healing of the graft at the fixation site, which typically takes several months. Rehabilitation protocols must initially prioritize protecting the graft to avoid compromising its healing process [68]. Patients follow a stepwise protocol, initially limiting their range of motion to protect the healing graft and gradually increasing it to safely restore knee flexibility. Central to rehabilitation is the re-establishment of knee strength and stability. Patients engage in targeted exercises to rebuild quadriceps, hamstring, and calf muscle strength, providing essential support for the healing knee joint [69]. Neuromuscular training is vital to enhance proprioception and coordination, reducing the risk of reinjury by conditioning muscles and ligaments to respond effectively to various movements and stresses [70]. Rehabilitation after PCL surgery can be mentally challenging, which emphasizes the need for psychological support and counseling to address anxiety, fear, or frustration related to the recovery process. Regular follow-up appointments with surgical teams or physical therapists are essential for progress monitoring and adjustments to ensure recovery stays on track [71]. Ultimately, the rehabilitation plan must be individualized, considering factors such as age, fitness level, and the presence of concomitant injuries to maximize its effectiveness in facilitating patient recovery [62].

## Conclusions

Arthroscopy-assisted fixation is a highly recommended method for treating avulsed PCL injuries, boasting several advantages over traditional open surgery. Its minimally invasive nature results in smaller incisions, reduced tissue disruption, and less postoperative pain, potentially translating to faster recovery. Its improved visualization allows for precise PCL reattachment, enhancing joint stability and clinical outcomes. Arthroscopy-assisted fixation matches the results of traditional open procedures. The effectiveness of this approach can vary according to the specific characteristics and the severity of the injury. Individually tailored to individual needs is important to ensure a successful recovery, and biomechanically, arthroscopy-assisted fixation restores essential knee stability and range of motion. Advancements in technology further improve patient outcomes, while the choice of graft material and surgical timing remain critical considerations. It is necessary to assess each case individually to determine the most appropriate treatment approach.
